# Clinical and epidemiological characteristics and outcomes of patients affected by COVID-19 in the Intensive Care Unit

**DOI:** 10.1590/0034-7167-2023-0527

**Published:** 2025-01-10

**Authors:** Priscila da Silva Timoteo, Kleyton Santos de Medeiros, Francis Solange Vieira Tourinho

**Affiliations:** I Universidade Federal de Santa Catarina. Florianópolis, Santa Catarina, Brazil; II Instituto de Ensino, Pesquisa e Inovação - Liga Contra o Câncer. Natal, Rio Grande do Norte, Brazil

**Keywords:** COVID-19, Disease Attributes, Epidemiologic Factors, Nursing Care, Intensive Care Units, COVID-19, Atributos de Enfermedad, Factores Epidemiológicos, Atención de Enfermería, Unidades de Cuidados Intensivos

## Abstract

**Objective:**

To understand the clinical and epidemiological characteristics, outcomes, and nursing care of adult patients affected by COVID-19 in the Intensive Care Unit.

**Methods:**

This is a quantitative, retrospective, and descriptive study. The study participants were clinical and epidemiological statistical reports. Variables analyzed included age, gender, race, comorbidities, signs and symptoms, length of hospital stay, use of mechanical ventilation, medications, infections, monitoring, invasive devices, positioning, diet, comfort, and clinical outcomes.

**Results:**

The majority of individuals were men, of white race, with a mean age of 63 years, hypertensive, diabetic, and obese. The average length of hospital stay was 16 days. Most required invasive mechanical ventilation, vasopressor drugs, sedoanalgesia, and neuromuscular blockers.

**Conclusion:**

Nursing care is related to monitoring, ventilation, medication administration, installation of devices, prone positioning, diet administration, and providing comfort.

## RESUMEN

**Objetivo:**

Conocer las características clínicas, epidemiológicas, desenlace y cuidados de enfermería de pacientes adultos afectados por COVID-19 en la Unidad de Cuidados Intensivos.

**Métodos:**

Estudio cuantitativo, retrospectivo y descriptivo. Los participantes del estudio fueron informes clínicos y epidemiológicos estadísticos. Se analizaron variables como: edad, género, raza, comorbilidades, signos y síntomas, período de hospitalización, uso de ventilación mecánica, medicamentos, infecciones, monitoreo, dispositivo invasivo, posicionamiento, dieta, confort y desenlace clínico.

**Resultados:**

La mayoría de los indivíduos eran hombres, de raza blanca, con una edad media de 63 años, hipertensos, diabéticos y obesos. El tiempo medio de hospitalización fue de 16 días. La mayoría utilizó ventilación mecánica invasiva, fármacos vasoactivos, sedoanalgesia y bloqueadores neuromusculares.

**Conclusión:**

Los cuidados de enfermería están relacionados con la monitorización, ventilación, administración de medicamentos, instalación de dispositivos, posicionamiento en prono, administración de dieta y confort.

## INTRODUCTION

COVID-19, caused by a coronavirus, emerged in a group of patients in December 2019 in the city of Wuhan, Hubei province, China^(1)^. With widespread transmissibility around the world, the WHO declared on January 30, 2020, that the disease was a Public Health Emergency of International Concern, the highest level of alert by the organization. Later, on March 11, 2020, COVID-19 was characterized as a pandemic^(2)^.

The increasing number of cases and the severity of the disease caused by the virus required countries to expand the number of Intensive Care Unit (ICU) beds^(3)^. However, the clinical management of critically ill patients with a COVID-19 diagnosis requiring intensive care is complex. Due to this complexity, the exponential growth of new cases, and the high demand for human and material resources, critically ill COVID-19 patients represent a significant challenge for care teams and healthcare systems^(4)^.

Studies describe how the disease presents clinically, but further studies and epidemiological data are needed due to the virus variants and the recent use of vaccines in the population—factors that can influence the clinical course of the disease.

Globally, several variants have been observed in patients infected with SARS-CoV-2, including Alpha, predominant in Europe and exclusively in North America; Beta, with higher transmissibility and rapid spread, which became dominant in South Africa; Gamma, discovered in Brazil, with higher transmissibility than the pre-existing variant; and Omicron, highly mutated, which emerged in South Africa^(5, 6, 7, 8)^.

The vaccines developed are based on three main strategies: using the entire microorganism, only portions of the microbe that stimulate the immune system, or just the genetic material that contains information for the production of specific proteins, rather than the whole virus^(9)^.

Nursing care for critically ill patients affected by COVID-19 during the pandemic is new, challenging, and complex due to the transmissibility and severity of the disease, the emergence of variants, and the development of vaccines.

Understanding the characteristics of hospitalized patients with COVID-19 provides healthcare professionals with information to guide practice and policy, prepare for future pandemic waves, understand the impact on our hospitals, identify areas for improvement in management, and enable international comparisons with other health jurisdictions and ongoing temporal assessments^(10)^.

## OBJECTIVE

To understand the clinical and epidemiological characteristics, outcomes, and nursing care of adult patients affected by COVID-19 in the ICU of a high-complexity public hospital.

## METHODS

### Ethical Aspects

The research follows the recommendations of Resolution No. 466, dated December 12, 2012, and Resolution No. 510, dated April 7, 2016^(^11,12^)^. It was approved by the Ethics Committee of the Hospital Institution and the Research Ethics Committee of the Federal University of Santa Catarina (UFSC) through the Brazil Platform. A waiver for the Informed Consent Form was requested. The study ensures confidentiality, guaranteeing the privacy of subjects with respect to the confidential data involved.

### Study Design, Period, and Location

This is a quantitative, retrospective, and descriptive study conducted in a medium-sized, high-complexity hospital in Florianópolis, SC, which is a reference in the state for specialties such as Internal Medicine, General Surgery, Neurology, Neurosurgery, and Orthopedics. The institution has 188 active beds, both clinical and surgical, of which 28 are ICU beds, with 14 exclusively for patients diagnosed with COVID-19. Data collection was carried out between February 26, 2020, and February 25, 2021.

### Sample, Inclusion, and Exclusion Criteria

Data were collected from the clinical and epidemiological statistical reports of patients diagnosed with COVID-19, admitted to the ICU of a hospital in Florianópolis (SC). The inclusion criteria were: clinical and epidemiological statistical reports of hospital patients aged 18 years and older, of both sexes, who were admitted to the ICU with a COVID-19 diagnosis confirmed by RT-PCR test, rapid test (RT) for SARS-CoV-2, or characteristic lung imaging observed in chest CT scans. The exclusion criteria were clinical and epidemiological statistical reports of hospital patients with inconclusive diagnoses, illegible records, or those without records of variables relevant to the study.

Data collection was carried out by the researcher herself, using clinical and epidemiological reports requested from the hospital’s statistics, epidemiology, and registry departments, based on the inclusion and exclusion criteria, provided without patient identification. The following variables were quantitatively analyzed: age, gender, race, pre-existing comorbidities, signs and symptoms at ICU admission, date and duration of hospitalization, type and duration of mechanical ventilation use, type and duration of vasopressor drug use, sedation and other medications, infections, monitoring, type and duration of invasive device use, duration of prone positioning, type and use of diet, hygiene, comfort, and clinical outcomes, including ICU discharge, hospital transfer, or death.

### Data Analysis and Statistics

Data analysis was performed using Microsoft Excel, through the grouping of demographic, clinical, intervention, and nursing care data provided to patients. Continuous variables are expressed as means, and categorical variables are expressed as percentages. Absolute (n) and relative (%) frequencies were used for qualitative variables, and measures of central tendency and dispersion for quantitative variables. The existence of associations was assessed using Pearson’s chi-square test. For the comparison of mean values, the Student’s t-test was used. The significance level used in the research was 5% (p<0.05). The Excel program was used to create the database, and the Stata 16.1 software was used for data analysis^(13)^.

## RESULTS

**Figure 1:**
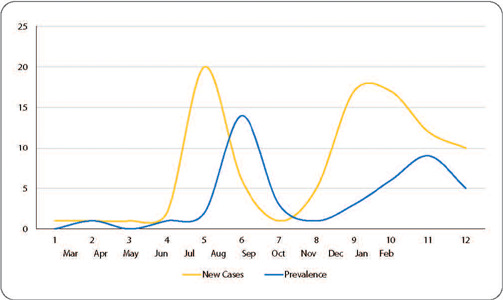
Number of new cases and prevalence of critically individuals hospitalized with COVID-19 from February 2020 to February 2021. Florianópolis, SC, Brazil

Examining the study period, the diagnosis of COVID-19, and the admission of individuals to the ICU, as shown in Figure 1, we observed that, in 2020, between March and June, there were very few cases. However, in July, there was an increase in the number of new cases of critically ill individuals with COVID-19. Between August and October, an additional increase in cases was also observed. Subsequently, between November and December, there was another fluctuation in the number of cases.

In January and February 2021, the number of critically ill individuals with COVID-19 remained stable. The months of August and December 2020, as well as January and February 2021, were characterized by a high prevalence of ICU admissions.

In this study, we described data from reports of 95 critically ill individuals affected by COVID-19. Of this total, examining the demographic characteristics, we found that 59 (62%) individuals were male and 36 (38%) were female. Regarding race, 78 (82.10%) of the individuals were white, 9 (9.47%) were black, 7 (7.36%) were mixed-race, and 1 (1.05%) was not reported. In terms of age, the individuals had an average age of 63 years, with a range from 26 to 89 years, representing the youngest and oldest ages, respectively. The demographic and clinical data can be seen in Table 1.

**Table 1:** Distribution of critically ill individuals affected by COVID-19 in a hospital, by gender, race, age, comorbidity, habits, and signs and symptoms. Florianópolis, SC, Brazil

Variables	n	(%)
Gender		
Male	59	62.10
Female	36	37.89
Race		
White	78	82.10
Black	9	9.47
Mixed-race	7	7.36
Not reported	1	1.05
Age		
18-40	8	8.42
41-60	23	24.21
61-80	58	61.05
81 and above	6	6.31
Comorbidities*		
Hypertension	56	58.94
Diabetes Mellitus	32	33.68
Obesity	32	33.68
Respiratory	16	16.84
Cardiac	5	5.26
Renal	7	7.36
Habits*		
Smoking	11	11.57
Former smoking	25	26.31
Alcohol use	5	5.26
Former alcohol use	2	2.10
No comorbidities	6	6.31
Signs and Symptoms*		
Dyspnea	76	80.00
Cough	37	38.94
Hyperthermia	20	21.05
Muscle pain	10	10.52
Diarrhea	10	10.52
Emesis	4	4.21

* *Individuals may present with more than one condition.*

More than half of the individuals had hypertension. Additionally, one-third had diabetes mellitus, followed by another third who were obese. Observing the habits, we noted a considerable number of former smokers, followed by current smokers. When analyzing the signs and symptoms presented by the individuals, we found that most had dyspnea, a little more than one-third had a cough, followed by hyperthermia.

The average length of ICU stay was 16.74 days, ranging from two to 68 days, corresponding to the shortest and longest hospital stays, respectively. Regarding the monitoring of individuals in the ICU, 83 (87.36%) had cardiac monitoring, oximetry, temperature, and invasive blood pressure monitoring, while 12 (12.63%) had cardiac monitoring, oximetry, temperature, and non-invasive blood pressure monitoring. Intensive nursing care during the hospitalization period and continuous monitoring of individuals were emphasized.

Of the study participants, 78 (82.10%) used an orotracheal tube (OTT), four (4.21%) were reintubated (ReOTT), 22 (23.15%) used a tracheostomy tube (TQT), 76 (80%) had a nasoenteral tube, 80 (84.21%) used a central venous catheter, 81 (85.26%) had invasive arterial pressure monitoring, 85 (89.47%) used an indwelling urinary catheter, nine (9.47%) had a hemodialysis catheter, 12 (12.63%) had peripheral venous access, ten (10.52%) had a chest drain, and one (1.05%) had an external ventricular drain. The most common nursing care included handling, installation, fixation, and guidance regarding invasive and non-invasive devices.

Table 2 describes the distribution of devices related to ventilation, the ventilatory modes used, the number of individuals, and the average usage in days. Devices such as tracheostomy tubes and orotracheal tubes had the highest average usage days among individuals, being 14 and 12 days, respectively. Of the total individuals, 79 (83.15%) used Invasive Mechanical Ventilation (IMV), with the ventilatory modes Volume Control (VC), Pressure Support (PS), and Pressure Control (PC), showing average usage of 8, 6, and 4 days, respectively. In this context, nursing plays a crucial role in care related to setting up the respiratory circuit, testing the mechanical ventilator, performing tracheal suctioning, and maintaining the patient’s head elevated.

**Table 2:** Distribution of devices related to ventilation, ventilatory mode used, and average usage in days by critically ill individuals affected by COVID-19. Florianópolis, SC, Brazil

Variables	n	Average usage in days
Device		
TOT	78	12
TQT	22	14
ReOTT	4	8
Nasal oxygen catheter	33	2.63
Large volume nebulization	15	5.26
Mask with reservoir	44	3.13
Ventilatory Mode		
VC	79	8.82
PC	50	4.65
PS	49	6.90
NIV	6	1.16

*Note: TOT (Orotracheal Tube); TQT (Tracheostomy); ReTOT(Orotracheal Reintubation); VC (Volume Control); PC (Pressure Control); PS (Pressure Support); NIV (Non-Unintentional Ventilation).*

The prone position was used by 39 (41%) individuals, with an average usage of 2 days. The self-prone position was performed by 33 (34.73%) individuals, with an average usage of 3 days. The types of vasopressor drugs, sedoanalgesia, and neuromuscular blockers (NMB) used, as well as the number of individuals and the average days of use, are described in Table 3. The main vasopressor drug used was norepinephrine. Regarding sedoanalgesia and neuromuscular blockers, fentanyl, propofol, midazolam, dexmedetomidine, and atracurium were the most commonly used by the individuals.

**Table 3:** Distribution of types of vasopressor drugs, sedoanalgesia, and neuromuscular blockers used, and average usage in days; types of antibiotics, antivirals, and antiparasitic drugs used by critically ill individuals affected by COVID-19. Florianópolis, SC, Brazil

Variables	n	(%)	Average usage in days
Vasoactive Drugs			
Norepinephrine	75	78.94	9.37
(Noradrenaline)			
Vasopressin	28	29.47	3.67
Sodium Nitroprusside	13	13.68	2.92
Nitroglycerin	7	7.36	1.85
Dobutamine	12	12.63	3.5
Sedoanalgesia / Neuromuscular Blockers			
Fentanyl	77	81.05	12.96
Propofol	77	81.05	12.68
Midazolam	77	81.05	9.86
Dexmedetomidine	52	54.73	7.63
Atracurium	50	52.63	6.94
Ketamine	28	29.47	8.75
Rocuronium	9	9.47	5.66
Antibiotics			
Ceftriaxone	72	75.78	...
Azithromycin	69	72.63	...
Piperacillin and	57	60.00	...
Tazobactam (Tazocin)			
Meropenem	55	57.89	...
Amikacin	24	25.26	...
Polymyxin	18	18.94	...
Teicoplanin	18	18.94	...
Sulfamethoxazole	18	18.94	...
Tigecycline	17	17.89	...
Vancomycin	12	12.63	...
Antivirals			
Oseltamivir	26	27.36	...
Ganciclovir	3	3.15	...
Hydroxychloroquine	2	2.10	...
Acyclovir	1	1.05	...
Antiparasitics			
Fluconazole	17	17.89	...
Albendazole	5	5.26	...
Metronidazole^(1)^	4	4.21	...
Micafungin	4	4.21	...

*Note: Conventional symbol used: ... Numeric data not available;^(1)^ Antibiotic and antiparasitic.*

Among the individuals, 43 (45.26%) had pulmonary infections, 27 (28.42%) had bloodstream infections, and 23 (24.21%) had urinary infections. These individuals were treated with multiple antibiotic regimens, as shown in Table 3. The main antibiotics used were ceftriaxone, azithromycin, piperacillin with tazobactam, meropenem, and amikacin. Additionally, oseltamivir and fluconazole were the most commonly used antiviral and antiparasitic agents, respectively.

The primary systemic corticosteroids used were dexamethasone and methylprednisolone, administered to 71 (74.73%) and 19 (20.00%) individuals, respectively. The most commonly used anticoagulants were enoxaparin and heparin, given to 61 (64.21%) and 14 (14.73%) individuals, respectively.

Given the critical condition of individuals with COVID-19 and the extensive use of medications, nursing care related to medication management involves consideration of indication, dosage, route, interaction, dilution, and administration.

Enteral nutrition was administered to 78 (82.10%) individuals, with an average duration of 16.43 days. Oral feeding was provided to 25 (26.31%) individuals, with an average duration of 3.6 days. However, there were no reports of total parenteral nutrition administration.

Due to the intensive and high-dependency care required, all 95 (100%) individuals received daily hygiene and comfort care, such as oral hygiene and bed baths, during their hospitalization period.

In describing the clinical and epidemiological characteristics, we can highlight that the main nursing care provided to critically ill adult patients with COVID-19 is related to monitoring, ventilation, medication administration, device installation, prone positioning, diet administration, hygiene, and comfort.

Regarding outcomes, of the total critically ill individuals affected by COVID-19, we observed that 49 (51.57%) progressed to death, 42 (44.21%) were discharged from the ICU, and 4 (4.21%) were transferred.

Analyzing the discharges and demographic characteristics, 26.31% of those discharged were male, 37.89% were white, and 22.10% were between 61 and 80 years old. On the other hand, among those who progressed to death, 31.57% were male, 41.05% were white, and 35.78% were between 61 and 80 years old. Upon examining the number of deaths and the main comorbidities of the patients, we identified that 35.78% had systemic arterial hypertension (SAH), 22.10% had diabetes mellitus (DM), and 8.42% were obese. Among the patients who were discharged, 18.94% had SAH, 10.52% had DM, and 9.47% were obese.

Observing the length of ICU stay, 29.47% of the patients were discharged in less than 17 days. However, 28.42% progressed to death within less than 17 days of ICU stay. Analyzing the outcomes and the use of invasive mechanical ventilation (IMV), 31.57% of the patients who used IMV were discharged. However, 45.26% of the patients who used IMV progressed to death.

## DISCUSSION

In July 2020, there was the highest and most significant number of new cases of critically ill individuals with COVID-19. Between August and October, there was a decrease in the number of cases. Following this, between November and December, there was another rise in the number of cases. In January and February 2021, there was a slight decline in the number of critically ill individuals with COVID-19. The months of August and December 2020, as well as January and February 2021, had the highest prevalence of ICU admissions.

The predominant epidemiological characteristics in our study are male gender, white race, and elderly individuals with an average age of 63 years. These findings are consistent with other studies, where the majority of patients were male^(14, 15)^, and the median age was also 63 years^(15, 16)^. Regarding race and ethnicity, one study showed that, out of 135 patients, 27 (20%) were black, and 9 (6.7%) were white^(15)^.

The most prevalent comorbidities among the individuals are hypertension, diabetes mellitus, and obesity. We highlight that one-third of the sample is classified as obese, and we note that slightly more than one-third of the individuals are classified as overweight. We observed other studies with similar percentage values. In a case series with 1,043 patients, 509 (49%) had hypertension^(14)^. Similarly, in this case-control study, hypertension (42%) and diabetes (20%) were the most frequent underlying diseases^(17)^. In this study, the rate of obese patients (BMI ≥ 30 kg/m^2^) was 1607/3935 (41%). The most common comorbidities were hypertension 2018/4197 (48%) and diabetes 1167/4196 (28%)^(16)^.

Regarding habits, the considerable number of former smokers, combined with the number of current smokers, represents more than one-third of the study population. This suggests that, despite some individuals having quit smoking, they are still at risk of developing severe forms of COVID-19.

In our study, we found that the most common signs and symptoms presented by the individuals were dyspnea, cough, and hyperthermia. A retrospective review conducted with 135 individuals admitted to the ICU describes fever (80.5%), cough (79.3%), and dyspnea (75.6%) as the main signs and symptoms presented^(15)^.

Regarding the average length of ICU stay, in the present study, the duration was almost double compared to other studies. A study conducted in Chicago reports that, of the 1,483 patients diagnosed with COVID-19 who required hospitalization, 528 (35.6%) were admitted to the ICU, with an average length of stay of 5 days^(18)^. In a study conducted in Italy, the average ICU stay was 9 days (n = 1,591)^(14)^.

The OTT was the device used by the majority of individuals, and IMV was the most significant support, followed by devices such as a mask with a reservoir, nasal oxygen catheter, and TQT. Similar numbers were found among 1,300 patients with available respiratory support data, where 1,287 (99%) required respiratory support, including 1,150 (88%) patients who needed endotracheal intubation and received mechanical ventilation^(14)^.

In our study, the prone position was necessary for almost half of the individuals, and self-proning was performed by more than one-third of them. However, the average duration of application in days was low. A systematic review reports that the application of the prone position alters the mechanics and physiology of gas exchange during ventilation, providing more consistent oxygenation in patients affected by COVID-19. Although the management is complex, it prevents worsening and brings clinical benefits^(19)^. Sixty percent (120/199) of healthcare professionals indicated that the prone position was used in more than 60% of their patients^(20)^. Training the multidisciplinary team to meet these new demands is extremely important for better patient management and the successful execution of the maneuver^(19)^.

In this study, almost half of the population presented with pulmonary infection, followed by nearly one-third with bloodstream infection, and just under one-third with urinary infection. The most commonly used antibiotics were classified as cephalosporins, macrolides, and broad-spectrum beta-lactams. Similar numbers were observed in a study conducted in Spain, which included 140 critically ill COVID-19 patients, where 30 episodes of lower respiratory tract infection were recorded, of which 21 were ventilator-associated pneumonia, 28 episodes of primary bloodstream infection, 24 episodes of catheter-related bloodstream infection, and 7 episodes of urinary tract infection. Most patients were treated with ceftriaxone after admission (n =120, 86%) and/or azithromycin (n = 118, 84%). Additionally, 105 patients (75%) received another antibiotic treatment for at least 3 days. A high proportion of patients also received corticosteroid therapy (90%)^(17)^.

Enteral feeding was administered to the majority of individuals, with a higher average usage. However, oral feeding was offered to just under one-third of individuals, with a lower average usage. There was no report of total parenteral nutrition administration. In an observational study, the most common type of enteral access device used was a smaller-gauge nasogastric tube. For patients infected with SARS-CoV-2, the placement of feeding tubes was more frequently performed by nurses^(20)^.

A study conducted in China describes that critically ill patients with COVID-19 are at risk of rapidly progressing to complications and require a high level of care from ICU nurses. Key points based on the literature about intensive nursing care for these patients are highlighted, including intubation and ventilation, prevention of venous thromboembolism (VTE), care for patients on Extracorporeal Membrane Oxygenation (ECMO), care for those requiring enteral nutrition, psychological support, and nursing management in the ICU for COVID-19 patients^(21)^.

A retrospective observational study, including 95 COVID-19 patients, conducted in five ICUs across three hospitals in Belgium, assessed the nurse-to-patient ratio using the Nursing Activities Score. Nursing activities such as monitoring and titration, mobilization, and hygiene care were higher because the patients were more critical, as indicated by a higher APACHE II score, the use of the prone position, and isolation measures, which require more direct care time^(22)^.

A cross-sectional observational study conducted with critically ill COVID-19 patients in Iran reports that patients had an average heart rate of 89.7 bpm, 13 patients experienced arrhythmias, and those who received antivirals had a longer QT interval^(23)^. A retrospective study with clinical data of SpO2 and SaO2 measurements from arterial blood gases of ICU patients with COVID-19, upon discharge or transfer, indicates that accurate clinical monitoring is crucial for ensuring patient safety and supporting management decisions. In this study, half of the sample presented with deep vein thrombosis or pulmonary embolism, with nearly all receiving anticoagulant treatment^(24)^.

Nursing care focused on hygiene and comfort, such as oral hygiene and bed baths, is extremely important for preventing infections and promoting health recovery. Oral hygiene is a crucial practice for maintaining health and comfort in intubated patients and those on mechanical ventilation, as it helps prevent ventilator-associated pneumonia, other complications, and reduces the length of hospital stay^(25)^.

Almost half of the population had discharge as their outcome, while the other half, unfortunately, had death as their outcome. These data show that our ICU mortality rate was high compared to other studies. Among the 1,581 patients with data available as of March 25, 2020, 256 (16%) were discharged from the ICU, and 405 (26%) died in the ICU^(14)^. The overall 90-day mortality was 31% and was higher in elderly, obese, diabetic, immunocompromised patients, and those with multiple organ dysfunction upon ICU admission^(16)^.

However, there was no significant difference in discharge or death outcomes concerning demographic characteristics, as well as the length of stay in the ICU. The majority of patients who died had hypertension (HAS) and required invasive mechanical ventilation (IMV). When evaluating the variables according to stratifications by sex, age group (adults: 26 to 59 years/elderly: 60 to 89 years), and patient hospitalization outcomes, there was no statistically significant association with any variable concerning sex.

In the age group variable, a statistically significant association (p<0.05) was found, as expected, with age, obesity comorbidity, and hospitalization outcome. Adults had a statistically higher frequency of obesity than the elderly (40.9% vs. 16.4%); conversely, the elderly showed a higher prevalence of age (71.0 years vs. 44.5 years) and death outcome (62.5% vs. 32.1%) compared to adults. In the hospitalization outcome variable, a statistically significant association (p<0.05) was found with the variables age, age group, and hypertension (HAS). Patients who died were more likely to be older (67.8 years vs. 57.9 years), elderly (81.6% vs. 55.8%), and have hypertension (70.8% vs. 50.0%) compared to those who were discharged.

### Study Limitations

This study was conducted at a single center. It is important to highlight the need for investments in information systems and electronic patient records, which are essential tools for generating reports and indicators that provide an overall clinical and epidemiological view of patient health services. When working with secondary data from patient records, the study presents limitations inherent to this type of source, such as retrospective information, lower reliability, and issues related to the accuracy of the records.

### Contributions to Nursing, Health, and Public Policy

In dealing with the pandemic, understanding the epidemiology of severe COVID-19 highlights the need for investments in public policies focused on adult health. Additionally, studies of this type contribute to improving nursing care in health services.

## CONCLUSIONS

This study made it possible to identify that severe COVID-19 predominantly affected male individuals, those of the white race, the elderly, and individuals with multiple comorbidities such as hypertension, diabetes mellitus, and obesity. Unfortunately, death was the outcome for half of the individuals. The main nursing care provided is related to monitoring, ventilation, medication administration, device installation, prone positioning, diet administration, hygiene, and comfort.

We emphasize the practical, technical-scientific knowledge of nurses due to the uniqueness, severity, and complexity of the disease, the patient’s hemodynamic instability, and multi-organ dysfunction, as well as the wide range of devices, monitoring, medications, positioning, and procedures used and/or performed.
