# Supplementary material for: Clinical and epidemiological characteristics and outcomes of patients affected by COVID-19 in the Intensive Care Unit
Source: Rev Bras Enferm. 2025 Jan 10;77(Suppl 1):e20230527. doi: 10.1590/0034-7167-2023-0527 (PMC11726807; doi:10.1590/0034-7167-2023-0527)
Supplement: Translation

# Translation: Características clínicas, epidemiológicas e desfecho de pacientes acometidos por COVID-19 em Unidade de Terapia Intensiva

**DOI:** 10.1590/0034-7167-2023-0527pt

**Authors:** Priscila da Silva Timoteo, Kleyton Santos de Medeiros, Francis Solange Vieira Tourinho

**Affiliations:** I Universidade Federal de Santa Catarina. Florianópolis, Santa Catarina, Brasil; II Instituto de Ensino, Pesquisa e Inovação - Liga Contra o Câncer. Natal, Rio Grande do Norte, Brasil

**Keywords:** COVID-19, Atributos de Doença, Fatores Epidemiológicos, Cuidados de Enfermagem, Unidade de Terapia Intensiva

## Abstract

**Objetivo:**

Conhecer as características clínicas, epidemiológicas, desfecho e cuidados de enfermagem a pacientes adultos acometidos por COVID-19 em Unidade de Terapia Intensiva.

**Métodos:**

Estudo quantitativo, retrospectivo e descritivo. Os participantes do estudo foram relatórios clínicos e epidemiológicos estatísticos. Foram analisadas variáveis como: idade, gênero, raça, comorbidades, sinais e sintomas, período de internação, uso de ventilação mecânica, medicamentos, infecções, monitoramento, dispositivo invasivo, posicionamento, dieta, conforto e desfecho clínico.

**Resultados:**

A maioria dos indivíduos eram homens, de raça branca, com idade média de 63 anos, hipertensos, diabéticos e obesos. A média de tempo de internação foi de 16 dias. A maioria fez uso de ventilação mecânica invasiva, drogas vasoativas, sedoanalgesia e bloqueadores neuromusculares.

**Conclusão:**

Os cuidados de enfermagem estão relacionados à monitorização, ventilação, administração de medicamentos, instalação de dispositivos, posicionamento em prona, administração de dieta e conforto.

## INTRODUÇÃO

A doença COVID-19, causada por um coronavírus, surgiu em um grupo de pacientes em dezembro de 2019, na cidade de Wuhan, província de Hubei, na China^(1)^. Com a transmissibilidade disseminada pelo mundo, a OMS declarou em 30 de janeiro de 2020 que a doença era uma Emergência de Saúde Pública de Importância Internacional, o mais alto nível de alerta da Organização. Posteriormente, em 11 de março de 2020, a COVID-19 foi caracterizada como uma pandemia^(2)^.

O crescimento do número de casos e a gravidade da doença causada pelo vírus exigiram que os países aumentassem a quantidade de vagas em Unidades de Terapia Intensiva (UTI)^(3)^. No entanto, o manejo clínico de pacientes críticos com diagnóstico de COVID-19 que necessitam de cuidados intensivos é complexo. Devido a essa complexidade, ao crescimento exponencial de novos casos e à alta demanda de recursos humanos e materiais, pacientes graves com COVID-19 representam um grande desafio para equipes assistenciais e para os sistemas de saúde^(4)^.

Estudos descrevem como a doença se apresenta na clínica, mas há necessidade de outros estudos e dados epidemiológicos devido às variantes do vírus e ao uso recente de vacinas na população, fatores que podem influenciar a clínica da doença.

Em todo o mundo, observaram-se variantes em pacientes infectados pelo SARS-CoV-2, como: Alfa, predominante na Europa e exclusivamente na América do Norte; Beta, de maior transmissibilidade e rápida disseminação, que se tornou dominante na África do Sul; Gamma, descoberta no Brasil, com maior transmissibilidade do que a variante pré-existente; e Omicron, fortemente mutante, que surgiu na Africa do Sul^(5, 6, 7, 8)^.

As vacinas desenvolvidas são baseadas em três estratégias principais, incluindo: todo o microrganismo, apenas porções do micróbio que estimulam o sistema imunológico, ou apenas os materiais genéticos que contêm informações para a produção de proteínas específicas, em vez de todo o vírus^(9)^.

O cuidado de enfermagem a pacientes críticos acometidos pela COVID-19 na pandemia é algo novo, desafiador e complexo, devido à transmissibilidade e gravidade da doença, ao surgimento de variantes e ao desenvolvimento de vacinas.

O conhecimento das características dos pacientes internados no hospital com COVID-19 fornece aos profissionais de saúde informações para orientar a prática e a política, preparar-se para futuras ondas pandêmicas, entender o impacto em nossos hospitais, identificar áreas para melhorias no gerenciamento e permitir comparações internacionais com outras jurisdições de saúde e avaliações temporais contínuas^(10)^.

## OBJETIVO

Conhecer as características clínicas, epidemiológicas, desfecho e cuidados de enfermagem a pacientes adultos acometidos por COVID-19 em Unidade de Terapia Intensiva.

## MÉTODOS

### Aspectos Éticos

A pesquisa segue as recomendações da Resolução nº 466, de 12 de dezembro de 2012, e da Resolução nº 510, de 7 de abril de 2016^(11, 12)^. Foi aprovada pelo Comitê de Ética da Instituição Hospitalar e pelo Comitê de Ética em Pesquisa da Universidade Federal de Santa Catarina (UFSC), por meio da Plataforma Brasil. Foi solicitada dispensa do Termo de Consentimento Livre e Esclarecido. O estudo conta com a garantia do sigilo, que assegura a privacidade dos sujeitos quanto aos dados confidenciais envolvidos.

### Desenho, Período e Local do Estudo

Trata-se de um estudo quantitativo, retrospectivo e descritivo, realizado em um hospital de médio porte e alta complexidade do município de Florianópolis/SC, referência no Estado nas especialidades de Clínica Médica, Cirurgia Geral, Neurologia, Neurocirurgia e Ortopedia. A instituição possui 188 leitos ativos, clínicos e cirúrgicos, dos quais 28 são de UTI, sendo 14 exclusivos para pacientes com diagnóstico de COVID-19. O período da coleta de foi realizada entre 26 de fevereiro de 2020 a 25 de fevereiro de 2021.

### Amostra, Critérios de Inclusão e Exclusão

Os dados foram coletados dos relatórios clínicos e epidemiológicos estatísticos de pacientes com diagnóstico de COVID-19, internados no setor de UTI de um hospital de Florianópolis (SC). Os critérios de inclusão foram: relatório clínico e epidemiológico hospitalar estatístico de pacientes maiores de 18 anos, de ambos os sexos, que estiveram internados no setor de UTI, com diagnóstico de COVID-19 confirmado por teste RT-PCR, teste rápido (TR) para SARS-CoV-2 ou imagem pulmonar característica observada em tomografia computadorizada de tórax. Os critérios de exclusão foram relatórios clínicos e epidemiológicos hospitalares estatísticos de pacientes com diagnósticos inconclusivos, registros ilegíveis ou que não possuíam registros das variáveis relevantes para o estudo.

A coleta de dados foi realizada pela própria pesquisadora, por meio de relatórios clínicos e epidemiológicos solicitados ao setor de estatística, epidemiologia e registro hospitalar, conforme os critérios de inclusão e exclusão, fornecidos sem identificação dos pacientes. Analisou-se o quantitativo das seguintes variáveis: idade, gênero, raça, comorbidades prévias, sinais e sintomas apresentados na admissão na UTI, data e período de internação, tipo e período de uso de ventilação mecânica, tipo e período de uso de drogas vasoativas, sedação e outros medicamentos, infecções apresentadas, monitoramento, tipo e período de uso de dispositivo invasivo, período de uso de posicionamento prona, tipo e uso de dieta, higiene, conforto e desfecho clínico, incluindo alta da UTI, transferência hospitalar ou óbito.

### Análise dos Resultados e Estatísticas

A análise de dados foi realizada com o auxílio do programa Microsoft Excel, através do agrupamento de dados demográficos, clínicos, intervenções e cuidados de enfermagem prestados aos pacientes. As variáveis contínuas são expressas como média, e as variáveis categóricas são expressas em porcentagem. Na descrição dos dados, foram utilizadas frequências absolutas (n) e relativas (%) para variáveis qualitativas, e medidas de tendência central e dispersão para as quantitativas. A existência de associação foi avaliada por meio do teste de qui-quadrado de Pearson. Para a comparação dos valores médios, foi utilizado o *t Student test.* O nível de significância usado na pesquisa foi de 5% (p<0,05). O programa Excel foi empregado para elaboração do banco de dados, e o software Stata 16.1, para análise dos dados^(13)^.

## RESULTADOS

Ao explorarmos o período de estudo, o diagnóstico de COVID-19 e a internação dos indivíduos na UTI, conforme apresentado na Figura 1, observamos que, no ano de 2020, entre os meses de março e junho, houve a ocorrência de pouquíssimos casos. Entretanto, no mês de julho, houve um aumento no número de casos novos de indivíduos críticos com COVID-19. Entre os meses de agosto e outubro, também se observou um aumento no número de casos. Logo após, entre os meses de novembro e dezembro, houve uma nova variação no número de casos.

**Figura 1:** Número de casos novos e prevalência de indivíduos críticos internados com CONVID-19 entre fevereiro de 2020 a fevereiro de 2021. Florianópolis, SC, Brasil

Nos meses de janeiro e fevereiro de 2021, observou-se uma manutenção no número de casos de indivíduos críticos com COVID-19. Os meses de agosto e dezembro de 2020, assim como janeiro e fevereiro de 2021, foram caracterizados pela prevalência de internações em UTI.

No estudo, descrevemos dados de relatórios de 95 indivíduos críticos acometidos por COVID-19. Desse total, ao tratar das características demográficas, verificamos que 59 (62%) indivíduos eram do gênero masculino e 36 (38%) do gênero feminino. Ao observar a raça, 78 (82,10%) dos indivíduos eram brancos, 9 (9,47%) eram pretos, 7 (7,36%) eram pardos e 1 (1,05%) não informado. Em relação à idade, os indivíduos apresentaram média de 63 anos, com intervalo de 26 a 89 anos, representando a menor e a maior idade, respectivamente. Os dados demográficos e clínicos podem ser observados na Tabela 1.

**Tabela 1:** Distribuição de indivíduos críticos acometidos por COVID-19 de um hospital em relação a gênero, raça, idade, comorbidade, hábito e sinais e sintomas. Florianópolis, SC, Brasil

Variáveis	n	(%)
Gênero		
Masculino	59	62,10
Feminino	36	37,89
Raça		
Branco	78	82,10
Preto	9	9,47
Pardo	7	7,36
Não identificado	1	1,05
Idade		
18-40	8	8,42
41-60	23	24,21
61-80	58	61,05
81 e mais	6	6,31
Comorbidade*		
Hipertensão Arterial	56	58,94
Diabetes Mellitus	32	33,68
Obesidade	32	33,68
Respiratória	16	16,84
Cardíaca	5	5,26
Renal	7	7,36
Hábito*		
Tabagismo	11	11,57
Ex-tabagismo	25	26,31
Etilismo	5	5,26
Ex-etilismo	2	2,10
Sem comorbidades	6	6,31
Sinais e sintomas*		
Dispneia	76	80,00
Tosse	37	38,94
Hipertermia	20	21,05
Dor muscular	10	10,52
Diarreia	10	10,52
Êmese	4	4,21

* *Os indivíduos podem apresentar mais de um (a).*

Mais da metade dos indivíduos apresentava hipertensão arterial. Além disso, um terço apresentava diabetes mellitus, seguido de um terço que apresentava obesidade. Observando os hábitos, verificamos um número considerável de indivíduos ex-tabagistas, seguido por indivíduos tabagistas. Ao analisarmos os sinais e sintomas apresentados pelos indivíduos, constatamos que a maioria apresentou dispneia, pouco mais de um terço apresentou tosse, seguida por hipertermia.

A média de tempo de internação em UTI foi de 16,74 dias, com intervalo entre dois e 68 dias, correspondendo ao menor e ao maior tempo de internação de um indivíduo, respectivamente. Em relação à monitorização dos indivíduos na UTI, 83 (87,36%) deles tiveram monitorização cardíaca, oximetria, temperatura e pressão arterial invasiva, enquanto 12 (12,63%) tiveram monitorização cardíaca, oximetria, temperatura e pressão não invasiva. Destaca-se o cuidado intensivo de enfermagem durante o período de internação e a monitorização contínua dos indivíduos.

Dos participantes do estudo, 78 (82,10%) fizeram uso de Tubo Orotraqueal (TOT), quatro (4,21%) foram reintubados (ReTOT), 22 (23,15%) fizeram uso de cânula de traqueostomia (TQT), 76 (80%) de sonda nasoenteral, 80 (84,21%) de cateter venoso central, 81 (85,26%) de pressão arterial invasiva, 85 (89,47%) de sonda vesical de demora, nove (9,47%) de catéter de hemodiálise, 12 (12,63%) de acesso venoso periférico, dez (10,52%) de dreno de tórax e um (1,05%) de derivação ventricular externa. Os cuidados de enfermagem mais utilizados incluíram a manipulação, instalação, fixação e orientações em relação a dispositivos invasivos e não invasivos.

A Tabela 2 descreve a distribuição de dispositivos relacionados à ventilação, o modo ventilatório utilizado, o número de indivíduos e a média de uso em dias. Os dispositivos como cânula de traqueostomia e tubo orotraqueal tiveram as maiores médias de uso em dias pelos indivíduos, sendo 14 e 12 dias, respectivamente. Do total de indivíduos, 79 (83,15%) fizeram uso de Ventilação Mecânica Invasiva (VMI), com os modos ventilatórios Volume Controlado (VC), Pressão de Suporte (PS) e Pressão Controlada (PC), apresentando médias de uso de 8, 6 e 4 dias, respectivamente. Neste contexto, a enfermagem desempenha um papel importante nos cuidados relacionados à montagem do circuito respiratório, testagem do ventilador mecânico, aspiração traqueal e manutenção da cabeceira elevada do indivíduo.

**Tabela 2:** Distribuição de dispositivos relacionados à ventilação, modo ventilatório utilizado e média de uso em dias pelos indivíduos críticos acometidos por COVID-19. Florianópolis, SC, Brasil

Variáveis	n	Média de uso em dias
Dispositivo		
TOT	78	12
TQT	22	14
ReTOT	4	8
Cateter Nasal de Oxigênio	33	2,63
Macronebulização	15	5,26
Máscara com Reservatório	44	3,13
Modo Ventilatório		
VC	79	8,82
PC	50	4,65
PS	49	6,90
VNI	6	1,16

*Nota: TOT (Tubo Orotraqueal); TQT (Traqueostomia); ReTOT (Reintubação Orotraqueal); VC (Volume Controlado); PC (Pressão Controlada); PS (Pressão de Suporte); VNI (Ventilação Não Insavisa).*

A posição prona foi utilizada por 39 (41%) indivíduos, com uma média de uso de 2 dias. Já a posição de autoprona foi realizada por 33 (34,73%) indivíduos, com uma média de uso de 3 dias. O tipo de droga vasoativa, sedoanalgesia e bloqueador neuromuscular (BNM) utilizado, assim como o número de indivíduos e a média de dias de uso, estão descritos na Tabela 3. A principal droga vasoativa utilizada foi a noradrenalina. Em relação à sedoanalgesia e bloqueador neuromuscular, fentanila, propofol, midazolam, dexmedetomidina e atracúrio foram os mais utilizados pelos indivíduos.

**Tabela 3:** Distribuição de tipos de droga vasoativa, sedoanalgesia e bloqueador neuromuscular utilizados e média de uso em dias; tipos de antibiótico, antiviral e antiparasitário utilizados pelos indivíduos críticos acometidos por COVID-19. Florianópolis, SC, Brasil

Variáveis	n	(%)	Média de uso em dias
Droga Vasoativa			
Noradrenalina	75	78,94	9,37
Vasopressina	28	29,47	3,67
Nitroprussiato de sódio	13	13,68	2,92
Nitroglicerina	7	7,36	1,85
Dobutamina	12	12,63	3,5
Sedoanalgesia / Bloqueador Neurouscular			
Fentanila	77	81,05	12,96
Propofol	77	81,05	12,68
Midazolam	77	81,05	9,86
Dexmedetomidina	52	54,73	7,63
Atracúrio	50	52,63	6,94
Ketamina	28	29,47	8,75
Rocurônio	9	9,47	5,66
Antibiótico			
Ceftriaxona	72	75,78	...
Azitromicina	69	72,63	...
Piperacilina e Tazocin	57	60,00	...
Meropenem	55	57,89	...
Amicacina	24	25,26	...
Polimixina	18	18,94	...
Teicoplanina	18	18,94	...
Sulfametoxazol	18	18,94	...
Tigeciclina	17	17,89	...
Vancomicina	12	12,63	...
Antiviral			
Oseltamivir	26	27,36	...
Ganciclovir	3	3,15	...
Hidroxicloroquina	2	2,10	...
Aciclovir	1	1,05	...
Antiparasitário			
Fluconazol	17	17,89	...
Albendazol	5	5,26	...
Metronidazol^(1)^	4	4,21	...
Micafungina	4	4,21	...

*Notas: Sinal convencional utilizado: ... Dado numérico não disponível; (1) antibiótico e antiparasitário.*

Dentre os indivíduos, 43 (45,26%) apresentaram infecção pulmonar, 27 (28,42%) apresentaram infecção em corrente sanguínea e 23 (24,21%) apresentaram infecção urinária. Estes fizeram uso de múltiplos esquemas de antibióticos, conforme apresentado na Tabela 3. Os principais antibióticos utilizados foram ceftriaxona, azitromicina, piperacilina com tazobactam, meropenem e amicacina. Além disso, oseltamivir e fluconazol foram o antiviral e o antiparasitário mais utilizados, respectivamente.

Os principais corticoides sistêmicos utilizados foram a dexametasona e a metilprednisolona, utilizados por 71 (74,73%) e 19 (20,00%) indivíduos, respectivamente. Os anticoagulantes mais utilizados foram a enoxaparina e a heparina, sendo administrados a 61 (64,21%) e 14 (14,73%) indivíduos, respectivamente.

Diante da criticidade dos indivíduos com COVID-19 e do uso de grande quantidade de medicamentos, observa-se que os cuidados de enfermagem relacionados aos medicamentos envolvem a indicação, dose, via, interação, diluição e administração.

A dieta enteral foi administrada em 78 (82,10%) indivíduos, com uma média de uso de 16,43 dias. A dieta por via oral foi oferecida a 25 (26,31%) indivíduos, com uma média de uso de 3,6 dias. No entanto, não houve relato de administração de dieta parenteral total.

Devido aos cuidados intensivos e de alta dependência, verificou-se que todos os 95 (100%) indivíduos receberam cuidados diários de higiene e conforto, como higiene oral e banho de leito, durante o período de internação.

Ao descrever as características clínicas e epidemiológicas, podemos destacar que os principais cuidados de enfermagem realizados a pacientes adultos críticos acometidos com COVID-19 estão relacionados aos eixos de monitorização, ventilação, administração de medicamentos, instalação de dispositivos, posicionamento prona, administração de dieta, higiene e conforto.

Em relação ao desfecho, do total de indivíduos críticos acometidos por COVID-19, observamos que 49 (51,57%) evoluíram para óbito, 42 (44,21%) receberam alta da UTI e 4 (4,21%) foram transferidos.

Analisando as altas e as características demográficas, 26,31% dos que receberam alta eram do sexo masculino, 37,89% eram de raça branca e 22,10% tinham idade entre 61 e 80 anos. Por outro lado, entre os que evoluíram para óbito, 31,57% eram do sexo masculino, 41,05% eram de raça branca e 35,78% tinham idade entre 61 e 80 anos. Ao verificar o número de óbitos e as principais comorbidades dos pacientes, identificamos que 35,78% apresentavam hipertensão arterial sistêmica (HAS), 22,10% tinham diabetes mellitus (DM) e 8,42% eram obesos. Dentre os pacientes que receberam alta, 18,94% apresentavam HAS, 10,52% tinham DM e 9,47% eram obesos.

Observando o tempo de internação em UTI, 29,47% dos pacientes evoluíram para alta com menos de 17 dias de internação. Entretanto, 28,42% evoluíram para óbito com menos de 17 dias de internação. Analisando o desfecho e o uso de ventilação mecânica invasiva (VMI), 31,57% dos pacientes que fizeram uso dela evoluíram para alta. No entanto, 45,26% dos pacientes que utilizaram VMI evoluíram para óbito.

## DISCUSSÃO

No mês de julho de 2020, houve o mais elevado e importante número de casos novos de indivíduos críticos com COVID-19. Entre os meses de agosto e outubro, houve queda no número de casos. Logo após, entre os meses de novembro e dezembro, houve uma nova elevação do número de casos. Nos meses de janeiro e fevereiro de 2021, houve uma leve queda no número de casos de indivíduos críticos com COVID-19. Os meses de agosto e dezembro de 2020 e janeiro e fevereiro de 2021 foram os meses em que houve maior prevalência de internação em UTI.

As características epidemiológicas predominantes no nosso estudo são indivíduos do gênero masculino, de raça branca e idosos com idade média de 63 anos. Esses dados estão de acordo com outros estudos, nos quais a maioria dos pacientes era do sexo masculino^(14, 15)^ e a idade mediana era idêntica, 63 anos^(15, 16)^. Em relação à raça e etnia, um estudo demonstrou que, de 135 pacientes, 27 (20%) eram pretos e 9 (6,7%) eram brancos^(15)^.

As comorbidades de maior prevalência entre os indivíduos são hipertensão arterial, diabetes mellitus e obesidade. Destacamos que um terço da amostra é classificado como obeso e alertamos que pouco mais de um terço dos indivíduos é classificado com sobrepeso. Observamos outros estudos com valores percentuais semelhantes. Em uma série de casos com 1.043 pacientes, 509 (49%) tinham hipertensão^(14)^. Da mesma forma, neste estudo de caso-controle, a hipertensão (42%) e o diabetes (20%) foram as doenças de base mais frequentes^(17)^. Neste estudo, a taxa de pacientes obesos (IMC ≥ 30 kg/m^2^) foi de 1607/3935 (41%). As comorbidades mais frequentes foram hipertensão 2018/4197 (48%) e diabetes 1167/4196 (28%)^(16)^.

Em relação aos hábitos, o número considerável de indivíduos ex-tabagistas, que, somado ao número de indivíduos tabagistas, representa mais de um terço da população de estudo. Isso sugere que, apesar de alguns indivíduos terem abandonado o hábito de fumar, eles correm o risco de desenvolver a forma grave da COVID-19.

No nosso estudo, constatamos que os sinais e sintomas mais apresentados pelos indivíduos foram dispneia, tosse e hipertermia. Uma revisão retrospectiva realizada com 135 indivíduos internados na UTI descreve febre (80,5%), tosse (79,3%) e dispneia (75,6%) como os principais sinais e sintomas apresentados^(15)^.

Em relação à média de tempo de internação em UTI, no presente estudo, o tempo foi quase o dobro comparado a outros estudos. Um estudo realizado em Chicago descreve que, dos 1.483 pacientes com diagnóstico de COVID-19 que precisaram de internação, 528 (35,6%) foram internados na UTI, com um tempo médio de permanência de 5 dias^(18)^. Já no estudo realizado na Itália, o tempo médio de permanência na UTI foi de 9 dias (n = 1.591)^(14)^.

O TOT foi o dispositivo utilizado pela maioria dos indivíduos e a VMI foi o suporte de maior importância, seguidos pelos dispositivos como máscara com reservatório, cateter nasal de oxigênio e TQT. Números semelhantes foram encontrados entre 1.300 pacientes com dados de suporte respiratório disponíveis, onde 1.287 (99%) precisaram de suporte respiratório, incluindo 1.150 (88%) pacientes que necessitaram de intubação endotraqueal e receberam ventilação mecânica^(14)^.

Em nosso estudo, a posição prona foi necessária para quase metade dos indivíduos, e a autoprona foi realizada por mais de um terço deles. No entanto, o tempo médio de aplicação em dias foi baixo. Uma revisão sistemática relata que a aplicação da posição prona altera a mecânica e a fisiologia da troca de gases na ventilação, proporcionando melhor consistência na oxigenação em pacientes acometidos por COVID-19. O manejo é complexo, mas previne o agravamento e traz benefícios clínicos^(19)^. Sessenta por cento (120/199) dos profissionais de saúde indicaram que a posição prona foi usada em mais de 60% de seus pacientes^(20)^. O treinamento da equipe multidisciplinar frente a essas novas demandas é extremamente importante para o melhor manejo do paciente e realização da manobra^(19)^.

Neste estudo, quase metade da população apresentou infecção pulmonar, seguida de quase um terço com infecção em corrente sanguínea e pouco menos de um terço com infecção urinária. Os antibióticos mais utilizados foram classificados como cefalosporina, macrolídeo e betalactâmico, de amplo espectro. Números próximos foram observados em um estudo realizado na Espanha, que incluiu 140 pacientes críticos com COVID-19, onde foram registrados 30 episódios de infecção do trato respiratório inferior, dos quais 21 foram pneumonia associada à ventilação mecânica, 28 episódios de infecção primária da corrente sanguínea, 24 episódios de infecção da corrente sanguínea relacionada a cateter e 7 episódios de infecção do trato urinário. A maioria dos pacientes foi tratada com ceftriaxona após a admissão (n = 120, 86%) e/ou azitromicina (n = 118, 84%). Além disso, 105 pacientes (75%) receberam outro tratamento com antibióticos por pelo menos 3 dias. Uma alta proporção de pacientes também recebeu terapia com corticosteroides (90%) ^(17)^.

O uso de dieta enteral foi administrado na maioria dos indivíduos, com uma média de uso maior. No entanto, a dieta por via oral foi oferecida a pouco menos de um terço dos indivíduos, com uma média de uso menor. Não houve relato de administração de dieta parenteral total. Em um estudo observacional, o tipo mais comum de dispositivo de acesso enteral utilizado foi um tubo nasogástrico de calibre menor. Para pacientes infectados com SARS-CoV-2, a colocação de sondas de alimentação foi realizada com mais frequência por enfermeiras^(20)^.

Um estudo realizado na China descreve que pacientes críticos com COVID-19 correm o risco de progredir rapidamente para complicações e exigem um alto nível de cuidado dos enfermeiros da UTI. São destacados pontos-chave baseados na literatura sobre cuidados intensivos de enfermagem realizados a esses pacientes, incluindo intubação e ventilação, prevenção de tromboembolismo venoso (TEV), cuidado em Oxigenação por Membrana Extracorpórea (ECMO), cuidado para os que requerem nutrição enteral, suporte psicológico e gerenciamento de enfermagem na UTI para pacientes com COVID-19^(21)^.

Um estudo observacional retrospectivo, incluindo 95 pacientes com COVID-19, realizado em cinco UTIs de três hospitais na Bélgica, avaliou a proporção enfermeiro-paciente usando o *Nursing Activities Score.* As atividades de enfermagem como monitoramento e titulação, mobilização e cuidados de higiene foram maiores pelo fato de os pacientes serem mais críticos, conforme indicado pelo escore APACHE II mais alto, pelo uso da posição prona e pelas medidas de isolamento, que exigem maior tempo de cuidado direto^(22)^.

Um estudo observacional transversal, realizado com pacientes críticos com COVID-19 no Irã, descreve que os pacientes tiveram uma frequência cardíaca média de 89,7 bpm, 13 pacientes apresentaram arritmia e quem recebeu antivirais apresentou intervalo QT mais longo^(23)^. Um estudo retrospectivo com dados clínicos de SpO2 e medições de SaO2 da gasometria arterial de pacientes internados em UTI com COVID-19, em condições de alta ou transferência, descreve que o monitoramento clínico preciso é fundamental para garantir a segurança do paciente e apoiar as decisões de gerenciamento. Neste estudo, metade da amostra apresentou trombose venosa profunda ou embolia pulmonar, sendo que quase a totalidade recebeu tratamento anticoagulante^(24)^.

Os cuidados de enfermagem em higiene e conforto, como a realização da higiene oral e banho de leito, são de extrema importância para a prevenção de infecções e reabilitação da saúde. A higienização oral é uma prática crucial para a manutenção da saúde e o conforto dos pacientes intubados e sob ventilação mecânica, prevenindo a ocorrência de pneumonias associadas à ventilação, outras complicações e diminuindo o tempo de internação^(25)^.

Quase metade da população teve como desfecho a alta, enquanto a outra metade, lamentavelmente, teve o óbito como desfecho. Esses dados demonstram que nossa taxa de mortalidade na UTI foi alta em relação a outros estudos. Entre os 1.581 pacientes, com dados disponíveis em 25 de março de 2020, 256 (16%) receberam alta da UTI e 405 (26%) morreram na UTI^(14)^. A mortalidade geral em 90 dias foi de 31% e foi maior em pacientes idosos, obesos, diabéticos, imunocomprometidos e naqueles com disfunção de múltiplos órgãos na admissão à UTI^(16)^.

Contudo, não houve diferença significativa no desfecho de alta ou óbito em relação às características demográficas, bem como em relação ao tempo de internação na UTI. O maior número de pacientes que foram a óbito apresentava HAS e fez uso de VMI. Ao avaliar as variáveis segundo as estratificações por sexo, faixa etária (adultos: 26 a 59 anos/idosos: 60 a 89 anos) e desfecho de internação do paciente, no caso do sexo não se verificou a existência de associação estatisticamente significativa com nenhuma variável.

Na variável faixa etária, verificou-se a existência de associação estatisticamente significativa (p<0,05), como esperado, com a idade, com a comorbidade obesidade e com o desfecho de internação. Os adultos apresentaram uma frequência estatisticamente maior de obesidade do que os idosos (40,9% vs 16,4%); em contrapartida, os idosos mostraram maior prevalência de idade (71,0 anos vs 44,5 anos) e do desfecho óbito (62,5% vs 32,1%) em comparação aos adultos. Na variável desfecho de internação, verificou-se a existência de associação estatisticamente significativa (p<0,05) com as variáveis idade, faixa etária e HAS. Os pacientes que foram a óbito apresentaram maior frequência de idade (67,8 anos vs 57,9 anos), de idosos (81,6% vs 55,8%) e de hipertensão (70,8% vs 50,0%) em comparação aos que tiveram alta.

### Limitações do Estudo

Estudo realizado em único centro. Cabe ressaltar a necessidade de investimentos em sistemas de informação e em prontuários eletrônicos de pacientes, que são ferramentas essenciais para gerar relatórios e indicadores que forneçam uma visão geral clínica do paciente e epidemiológica do serviço de saúde. Ao trabalhar com dados secundários de prontuários de pacientes, o estudo apresenta limitações inerentes a esse tipo de fonte, como informações retrospectivas, menor confiabilidade e problemas relativos à precisão nos registros.

### Contribuições para a Área da Enfermagem, Saúde e Política Pública

No enfrentamento da pandemia, o conhecimento da epidemiologia da forma grave da COVID-19 destaca a necessidade de investimentos em Políticas Públicas voltadas para a Saúde do Adulto. Além disso, estudos desse tipo contribuem para aprimorar o cuidado de enfermagem em serviços de saúde.

## CONCLUSÕES

Com este estudo, foi possível identificar que a COVID-19 grave acometeu predominantemente indivíduos do gênero masculino, de raça branca e idosos, bem como indivíduos com multicomorbidades, como hipertensão arterial, diabetes mellitus e obesidade. Lamentavelmente, o óbito foi o desfecho em metade dos indivíduos. Os principais cuidados de enfermagem realizados estão relacionados aos eixos de monitorização, ventilação, administração de medicamentos, instalação de dispositivos, posicionamento prona, administração de dieta, higiene e conforto.

Destacamos o conhecimento técnico-científico prático do enfermeiro devido à singularidade, gravidade e complexidade da doença, à instabilidade hemodinâmica e à disfunção multiorgânica do indivíduo, bem como à ampla gama de dispositivos, monitorização, medicamentos, posicionamento e procedimentos utilizados e/ou realizados.
